# Optimizing load scheduling and data distribution in heterogeneous cloud environments using fuzzy-logic based two-level framework

**DOI:** 10.1371/journal.pone.0310726

**Published:** 2024-12-13

**Authors:** Bei Cheng, Dongmei Li, Xiaojun Zhu

**Affiliations:** 1 Research Center for Educational Evaluation and Inspection, China National Academy of Educational Sciences (CNAES), Beijing, China; 2 School of Computer and Big Data, Minjiang University, Fuzhou, Fujian, China; Air University, PAKISTAN

## Abstract

Cloud environment handles heterogeneous services, data, and users collaborating on different technologies and resource scheduling strategies. Despite its heterogeneity, the optimality in load scheduling and data distribution is paused due to unattended requests for a prolonged time. This article addresses the aforementioned issue using a Two-level Scheduling and Distribution Framework (TSDF) using Fuzzy Logic (FL). This framework houses different fuzzification processes for load balancing and data distribution across different resource providers. First, the fuzzification between regular and paused requests is performed that prevents prolonged delays. In this process, a temporary resource allocation for such requests is performed at the end of fuzzification resulting in maximum waiting time. This is the first level optimality determining feature from which the second level’s scheduling occurs. In this level, the maximum low and high delay exhibiting distributions are combined for joint resource allocations. The scheduling is completely time-based for which the cumulative response delay is the optimal factor. Therefore, the minimum time-varying requests observed in the second level are fuzzified for further resource allocations. Such allocations follow the distribution completed intervals improving its distribution (13.07%) and reducing the wait time (7.8%).

## 1 Introduction

Cloud computing offers immediate access to a shared collection of customizable computer resources, such as networks, servers, storage, and services. These resources can be quickly allocated and released with minimal maintenance required. Fog computing expands the concept of cloud computing to the periphery of the network, enabling processing, storage, and networking services to be located in closer proximity to end devices and data sources. A fog-cloud environment is a system that integrates the cloud and fog computing frameworks, utilizing both the centralized cloud resources and dispersed fog nodes at the edge. This allows for data processing and analysis to occur in proximity to the origins, resulting in decreased latency and bandwidth requirements, while also taking advantage of cloud computing for resource-intensive computational jobs, processing large amounts of data, and storing data for extended periods of time. Hybrid fog-cloud models are becoming increasingly important for applications that require low latency, such as Internet of Things (IoT), smart cities, and real-time data analytics. By integrating fuzzy logic, cloud and fog systems are capable of managing uncertainty, imprecise data, and making intelligent choices to optimize resource allocation, scheduling, load distribution, and meeting quality of service (QoS) demands.

Fuzzy optimization is a process that is mainly used for the decision-making process. Fuzzy optimization uses minimum values which are presented in the database. Fuzzy optimization reduces the complexity of the decision-making process [1]. By offering a versatile and adaptable method to deal with uncertainties, complicated linkages, trade-offs, and hazards, fuzzy optimization has an impact on making decisions in cloud computing management of resources. Because workloads in cloud environments are dynamic and change over time, dynamic cloud environments allow for more efficient distribution of resources and load balancing. In cloud computing, issues related to load balancing and resource allocation can be specifically addressed via fuzzy optimization. Fuzzy optimization aids in capturing and simulating these nuanced relationships, allowing the model to take into account the complex interplay among resources as well as requests when making decisions. Fuzzy optimization is used for data distribution in cloud environments. Data distribution is a function that distributes necessary variables and information to a particular task [2]. Fuzzy optimization is commonly used in data distribution to reduce the latency and workflow ratio of the systems. The fuzzy-optimized data management (FDM) technique is used in a cloud-based environment [3]. The FDM technique is mainly used to extract the necessary information from a cloud database. The extracted information provides relevant data for the data distribution process [4]. The FDM-based optimization process increases the effectiveness and feasibility range of cloud computing systems [5]. The risks and problems which are occurred in optimization are identified using prediction methods. The problems are classified based on security risk management that reduces the latency in the computation process [6].

Load balancing is a process that distributes the necessary resources to perform a particular task in an application or system. Load balancing is an important task to perform in every cloud environment [7]. Load-balancing solutions are used to solve the problems which are presented during performing a task. The load balancing solution optimizes the response time of the task which reduces the energy consumption in the task optimization process [8]. An efficient load-balancing approach is used in a cloud computing environment. A fuzzy optimization algorithm is implemented in the balancing approach which balances the cloud based on the functions [9]. The fuzzy algorithm schedules the resources which are required to perform tasks in the cloud environment. The efficient load-balancing approach improves the overall load-balance range of the environments [10]. A clustering-based multiple objective dynamic load balancing technique (CMODLB) is used to balance the load in cloud computing systems. The clustering method organizes the load using a neural network that minimizes the latency in the balancing process. The CMODLB technique understands the cloud criteria which enhances the performance and efficiency range in the load-balancing process [11, 12].

Resource scheduling is a process that schedules the resources to perform a particular task in an application. Resource scheduling is used for data distribution and load-balancing processes in cloud environments [13]. The main goal of resource scheduling is to schedule the resources and data based on task requirements. The exact requirements of the tasks are identified which produces relevant information for the resource scheduling process [14]. The actual internal and external factors of tasks are also detected which maximizes the accuracy in resource scheduling for load balancing and data distribution processes [15]. The evolutionary computation (EC) algorithm is used for resource scheduling. The EC algorithm schedules the resources for data distribution that improves the quality of service (QoS) for the users in cloud environments [16]. The EC algorithm improves the real-time performance range of resource scheduling that provide optimal services to the users [17]. An efficient resource scheduling algorithm is also used to analyze the relevant datasets for the data distribution process. The resource scheduling algorithm provides specific data which are necessary for data distribution. The resource scheduling algorithm also manages the load balancing range in cloud computing systems [11, 18]. The problem is to determine the most efficient assignments of tasks to resources, with the goal of maximizing resource usage and decreasing response times, latencies, and imbalances across the diverse infrastructure. It is important to take into account factors such as fluctuating workloads, interdependencies between data, the additional time and effort required for communication, and the varying performance characteristics of different resources. The purpose is to create intelligent strategies for scheduling loads and distributing data that can adjust to variations in system diversity, quickly changing patterns of workloads, and fluctuations in resource availability, in order to fulfill quality of service criteria and achieve performance objectives. Efficient solutions must carefully manage the computational workloads and distribute data in a manner that prevents overcrowded areas, unused resources, unnecessary data transfers, and points of congestion, while making the best use of the diverse capabilities of cloud resources. The contributions of the article are disclosed below:

1. Proposing and designing a data distribution and load balancing framework for improving the request processing rate and reducing its pause rate across various user demands
2. Incorporating the fuzzy process for classifying and detecting requests based on wait time for consenting load handling and confining response delay
3. Performing an experimental validation for verifying the proposed framework’s efficiency through comparative analysis

Then the rest of the paper is organized as follows: Related Works section describes about the various researchers opinion regarding the load balancing process in cloud environment. Two-level Scheduling and Distribution Framework (TSDF) using Fuzzy Logic (FL) section describes the working process of data distribution and load balancing framework. Then the excellence of the system is evaluated in Results and Discussion section. At last is the Conclusion section.

## 2 Related works

Dahan et al. [19] developed a multi-agent ant colony optimization (ACO) algorithm for cloud service composition (CSC). The main aim of the algorithm is to solve CSC problems that are presented in cloud environments. ACO algorithm is mainly used here to identify the CSC issues and produce relevant solutions to the systems. The developed algorithm reduces the complexity level in cloud computing systems. The developed algorithm increases the quality of service (QoS) range of cloud systems. The algorithms performance could be highly dependent on specific parameter configurations.

Sun et al. [20] proposed a contract-based resource-sharing technique for task scheduling in fog-cloud environments. The scheduling technique is done based on the functional domain which mitigates the fog nodes. A spectral clustering method is used in the technique to select critical nodes for the scheduling process. The proposed technique reduces the overall time consumption ratio in the task scheduling process. The proposed technique improves the robustness and mobility level of fog-cloud environments. The effectiveness of the system depends on the amount of the resources scheduled.

Cho et al. [21] introduced a resource allocation control engine with reinforcement learning (RACER) for edge cloud systems. The actual goal of the approach is to reduce the response time of the systems. RACER is mainly used to solve the problems which are occurred during the workload data distribution process. The introduced allocation strategy provides an optimal solution to solve the issues in cloud environments. The introduced RACER strategy increases the quality of service (QoS) range in the data distribution process. The RACER performance evaluation is a complex process to be executed in a real and application specific environment.

Kchaou et al. [22] designed a particle swarm optimization (PSO) based task scheduling method for cloud workflows. The fuzzy clustering method interval type-2 fuzzy c-means (IT2FCM) is implemented in the method to reduce the movement in a workflow. PSO algorithm is mostly used to minimize the complexity ratio in a particular task or function. The designed scheduling method improves the efficiency and realisability range of workflow in cloud systems.

Qin et al. [23] proposed a budget-constrained adaptive iterated local search (AILS) framework in cloud environments for workflow scheduling. The proposed AILS framework is used to guide the search for cloud nodes. The AILS framework also provides a feasible solution to solve the problems which are faced by the cloud environment. AILS provides an optimal control scheme for cloud environments. Experimental results show that the AILS framework increases the robustness level of the systems. There may be difficulties in large-scale situations that are not apparent in smaller ones.

Mokni et al. [24] introduced a multi-objective fuzzy approach for workflow task scheduling in fog-cloud computing. The multi-objective optimization technique is used here to understand the exact requirement of a task. The introduced approach provides optimal resources and data to perform tasks in cloud computing systems. The introduced fuzzy approach reduces the complexity level in the task scheduling process. Fog-Cloud systems can have complex, diversified utilisation of resources.

El Motaki et al. [25] designed a weighted fuzzy c-means clustering approach for workload monitoring in a cloud environment. The designed approach is mainly used for anomaly detection. Abnormal behaviors, patterns, and datasets are detected from the database. The designed approach uses resource usage details which produce optimal data for the clustering approach. The designed approach reduces the latency in workflow which improves the significance range of cloud environments. The process of detection is off-line, which has limitations when real-time detection is necessary.

Zatwarnicki et al. [26] proposed a two-level fuzzy-neural load distribution strategy for cloud-based web systems. The main aim of the strategy is to reduce the response time in providing service to the users. The actual service time and cost of the tasks are reduced using a distribution strategy. The load distribution strategy also reduces the computational cost range in web-based applications. The proposed strategy minimizes the traffic ratio in the workflow that enhances the efficiency level of the systems. The system needs more computation power than other algorithms was a limitation factor.

Sun et al. [27] developed a dynamic inertia particle swarm optimization (DI-PSO) algorithm-based task scheduling for cloud service systems. The developed DI-PSO is implemented to realize the necessity of tasks in service systems. A normal distribution selection method is used in the approach which selects the feasible nodes for the scheduling process. The developed technique increases the accuracy in task scheduling that improves the quality of service (QoS) range of the cloud systems. It’s critical to assess whether these advantages need more resource use or additional performance trade-offs.

Farid et al. [28] introduced a multi-objective scheduling algorithm using fuzzy resource utilization (FR-MOS) for cloud environments. The introduced FR-MOS is used for workflow task scheduling processes in cloud-based systems. The important qualities and features of tasks are identified that provide relevant data for the scheduling process. The introduced FR-MOS technique maximizes the efficiency and performance level of cloud environments. The workflow of makespan metric was low with improved cost due to the reliability constraint.

Kang et al. [29] proposed an online scheduling algorithm for cloud environments. A big data analysis is implemented in the algorithm which analyzes the necessary datasets for further processes. The main aim of the algorithm is to improve the perception and functionality range of the task scheduling process. The proposed algorithm improves the effectiveness of the job satisfaction process. The proposed algorithm improves the quality of service (QoS) level of cloud-based environments. For QoS, it’s crucial to guarantee equitable resource distribution and prevent resource bottlenecks.

Yang et al. [30] designed a flexible resource scheduling for software-defined cloud manufacturing (SDCM) systems. Software-defined network (SDN) model is used in the method for reconfiguration and evaluation process. The time-sensitive data traffic problems are detected. Proper solutions are provided to solve the traffic problems that minimize the complexity range in SDCM systems. The designed scheduling method reduces the overall latency level in the computation process. Changes in automation and control logic could call for training and adaption.

Wang et al. [31] introduced a many-objective cloud manufacturing service selection using adaptive environment selection (MaOEA-AES) for cloud systems. The main aim of the method is to construct an optimal mechanism for the service scheduling process. The AES is mainly used here to schedule the service based on priorities and resources. The introduced MaOEA-AES improves the performance and efficiency range of cloud manufacturing systems. Due to the huge objective space, the suggested algorithm’s search procedure takes a long time.

Guo et al. [32] proposed a multi-objective task scheduling method using a fuzzy self-defense algorithm for cloud computing systems. A scheduling optimization strategy is used here to understand the exact content of the tasks. The scheduling optimization reduces both time and energy consumption levels in the computation process. The proposed scheduling method maximizes the accuracy in task scheduling that increase the quality of service (QoS) level of the systems. It is crucial to comprehend the algorithm’s internal mechanisms and variables in order to evaluate its efficacy and adjust it for various conditions.

Mokni et al. [33] proposed scheduling as a multi-object optimization issue in order to produce a planning solution that strikes a balance between reaction time, cost, and make-span to describe Internet of Things (IoT) process. To allocate time for a set of dependent IoT tasks modelled as a workflow. The results demonstrated that make-span with gain was 21.38% in contrast to fog and cloud as a main QoS metric. Guaranteeing compatibility and interoperability with various Internet of Things (IoT) systems is lacking.

Shukla et al. [34] presented a novel approach for scheduling workflows in a fog-cloud environment using Multi-objective Artificial Algae (MAA) technique. The identified task lists are applied for prioritizing the most workflow with high speed. The weighted sum based multi-objective function was adopted to an appropriate use of fog resources. The result findings provides improvement with 43% of execution time,28% of energy consumption rate and finally 10% high in overall cost. The system is limited to scheduling system that must be properly understood and used by user defined parameters and management.

Mahmoud et al. [35] introduced HLFR (Hybrid Load Factor and Response Time) technique for job scheduling in heterogeneous cloud computing systems, aimed at improving load balancing efficiency. The technique seeks to enhance resource usage and minimize task response times by taking into account both the load factor and reaction time of virtual machines during job assignment. The process comprises of two primary stages—the initial stage involves evaluating virtual machines according to their load factors, while the subsequent stage involves selecting the virtual machine with the lowest reaction time from the group of lightly loaded VMs discovered in the first stage. Simulation experiments comparing HLFR with existing algorithms such as Round Robin and Throttled load balancing demonstrated that HLFR outperformed in terms of average response time, degree of imbalance among virtual machines, and number of migrations needed to balance the load across the cloud system.

Lin et al. [36] proposes two new task scheduling algorithms designed for heterogeneous cloud computing environments aimed at improving system performance and resource utilization. The first is the Main Resource Load Balancing (MRLB) algorithm which considers both CPU and RAM usage on virtual machines when making scheduling decisions to achieve balanced load across the main resources. The second is the Time Balancing (TB) algorithm which focuses on minimizing total task completion time by assigning tasks to the fastest available virtual machine that can meet resource requirements. The algorithms were evaluated through simulations comparing them to traditional algorithms like Round Robin and were shown to outperform the traditional approaches in metrics like average task response time, degree of load imbalance across VMs, and resource utilization rates in heterogeneous cloud setups. The proposed algorithms provide effective load balancing and scheduling approaches tailored to the characteristics of modern heterogeneous clouds.

As discussed above the existing algorithms faces limitations like high waiting time, limited cloud resources especially in real-world deployments in selecting optimal features, complex load distribution, load handling dependency on parameter configurations.

In traditional load scheduling and distribution, the wait time is high and also resource allocation is very difficult to determine optimality features; therefore, the proposed framework requires high computational time to identify the optimal output for sequential load scheduling and data distribution. To solve these addressed issues and to augment the performance of fuzzification, TSDF is proposed for performing such types of requests in the cloud platform and scheduling is completely time-based. In the existing work, TSDF used load scheduling and data distribution for joint resource allocation and services where it accurately determines the load handling rate for individual resource providers with less wait time. This process helps TSDF to improve the performance of load balancing and data distribution for sequential resource allocation. Hence, the proposed TSDF-FL model provides better resource allocation and services to identify the paused requests to perform load scheduling and distribution with less computational time.

## 3 Two-level Scheduling and Distribution Framework (TSDF) using Fuzzy Logic (FL)

**Fig 1:**
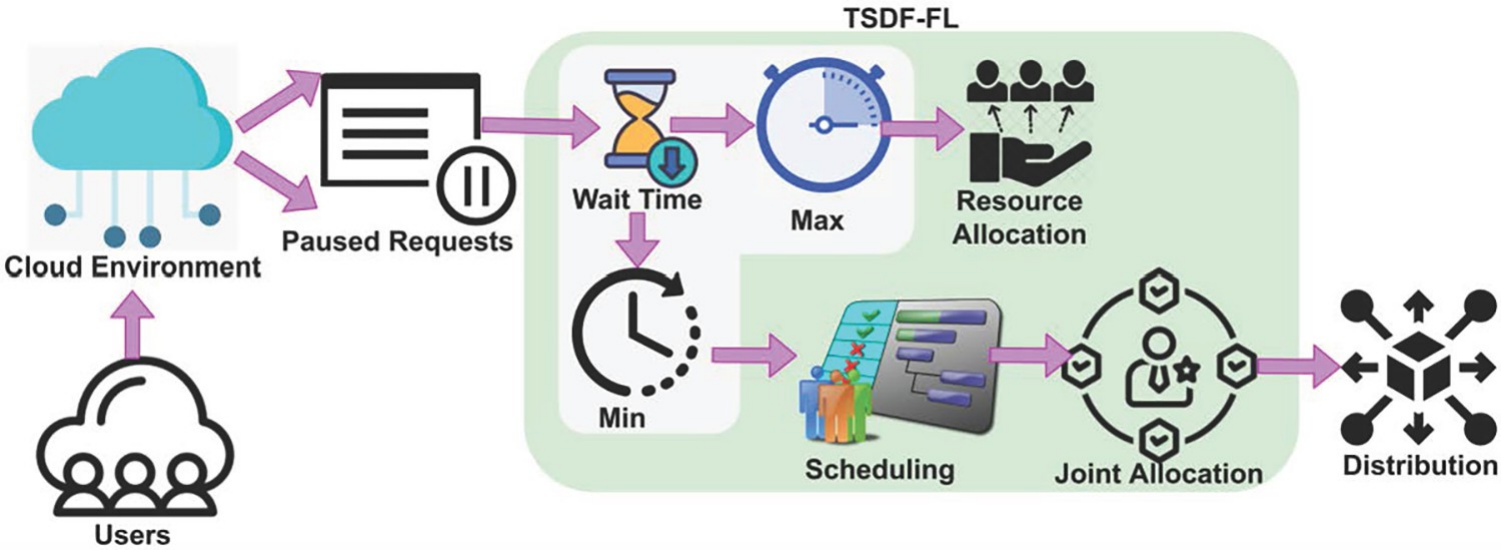
Proposed TSDF-FL Illustration.

Then, TSDF identifies the pending requests from the cloud environment if wait time *WT* does not satisfy the joint resource allocation. Two-Level Scheduling and Distribution Framework (TSDF-FL) model appears to be following a centralized scheduling and load balancing approach in cloud environments. Subsequently, TSDF computes the scheduling and joint resource allocation (i.e., optimality determining feature) with the help of two-level scheduling and distribution framework. If the resource allocation for the initial request is lesser than the resource allocation of paused requests, then TSDF segregates the paused requests for scheduling and joint allocation. Contrary to other cloud environment resource allocation and service analyses, the proposed TSDF-FL model utilizes the TSD framework to identify the aforementioned issues in load balancing and data distribution across different resource providers in the cloud environment as it can deal with high cumulative response delay and wait time. In this framework, TSDF is quite different because it does not rely on any restrictive conditions of the model and it provides optimal factors for resource allocation and services. Besides the TSDF-FL model employed, the fuzzification between regular and pursued requests observed from the cloud users is processed contrary to other fuzzification processes because it handles high cumulative response delay and unattended requests processing for a prolonged time. Furthermore, the fuzzification has an inbuilt adaptability nature with other models to process efficiently and the speed of the fuzzy logic is very high for identifying the global optimal solution for resource allocation and services. Therefore, the fuzzification helps the TSDF to improve its data distribution and to reduce the wait time for the sequence of request processing as compared to traditional work. Fig 1 presents the proposed TSDF-FL processes for load balancing and data distribution.

The TSDF-FL model applied two optimization algorithms to attain maximum load balancing and data distribution. In the first level, the TSDF-FL model first identifies a temporary resource allocation for regular and pursued requests and is verified at the end of fuzzification with minimal delay and minimal wait time using the TSDF. Then, the TSDF-FL model determines optimality features in the first level whereas in the second level resource scheduling occurs. In this level, the continuous load balancing and data distribution satisfies maximum high or low delay exhibiting distributions combined for joint resource allocations, and services are performed through time-varying requests observed from the second level. This performance helps the TSDF-FL model to improve the resource allocation and services for load balancing and data distribution at different resource providers. As a result, the TSDF-FL model increases the fuzzification process of continuous resource allocation and scheduling. The TSDF-FL model is briefly described in the following subsections.

### 3.1 Scheduling and distribution

The TSDF-FL model is used to handle heterogeneous services, users, and data observed from various technologies and resource scheduling for identifying and classifying the normal requests and paused requests in a cloud environment. Contrarily, the proposed framework selects the random value of *X* from the range [0,1] and then checks that the picked value *P* less than the request processing time *T*. If the above condition is satisfied by the first request, then TSDF allocates resource and provide services for a continuous distribution. Otherwise, TSDF reduces the wait time and response delay and subsequently performs joint resource allocation. This process of TSDF is performed continuously until the maximum wait time *WT* satisfies the joint resource allocation

Let us consider that the cloud environment consists of a set of users indicated as *CE*^*u*^ = {*CE*^1^, *CE*^2^,…,*CE*^*U*^}. To process the regular requests for load balancing and data distribution sequence across different resource providers. The TSDF randomly performs the normal requests and pursued requests from the cloud environment and considered for which the initial output. The paused requests exactly compute the data distribution at cloud computing with less wait time utilization. Consider that the regular request *rq*_*r*_ for load balancing and *ρ*(*rq*_*p*_)_*i*_ is the probability of paused requests occurring during the sequential resource allocation. From that, the optimality Δ of determining feature is expressed as

$$\Delta =FZ\sum_{WT}{rq}_{p_{i}}ln\;\rho {\left({rq}_{p}\right)}_{i}$$ (1)

And,

$$FZ(\Delta )=\frac{1}{WT}\{\sum_{{CE}^{u}=1}^{T}{rq}_{r}(T)+{rq}_{p}(T)-{Rs}_{dT}\}$$ (2)

Eq (1) and (2) computes *FZ* as a constant fuzzification process and the logarithm is the delay-less response. The changes in determining features Δ*C* due to maximum wait time, which is computed using

$$\Delta C=\sum_{WT}D[{rq}_{p_{i}},\rho {\left({rq}_{p}\right)}_{i}]-I{REA}_{Loc}$$ (3)

Where the sequential resource allocation is represented as in Eq (3) and (4)

$$\begin{array}{c} {{REA}_{Loc}}_{1}=FZ[{CE}^{1}]-{WT}_{i-1}\times D{\left[{rq}_{p_{i}},\rho {\left({rq}_{p}\right)}_{i}\right]}_{1}\\ {{REA}_{Loc}}_{2}=FZ[{CE}^{2}]-{WT}_{i}\times D{\left[{rq}_{p_{i}},\rho {\left({rq}_{p}\right)}_{i}\right]}_{2}\\ \vdots \\ {{REA}_{Loc}}_{i}=FZ{\left[{CE}^{U}\right]}_{i}-\Delta_{i}\times D{\left[{rq}_{p_{i}},\rho {\left({rq}_{p}\right)}_{i}\right]}_{i} \end{array}\}$$ (4)

**Fig 2:**
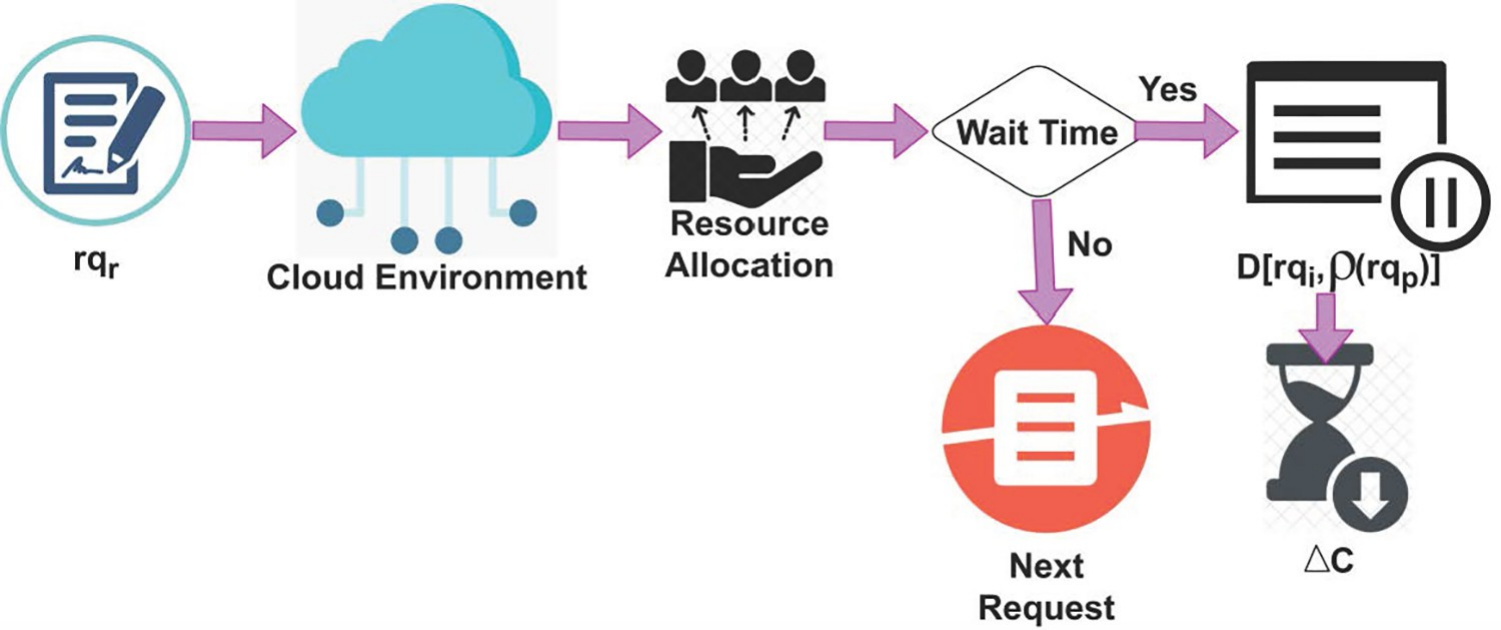
Changes detection for different wait times.

Where, the variable $D\left[{rq}_{p_{i}},\rho {\left({rq}_{p}\right)}_{i}\right]$ denotes different requests from the initial cloud environment used for load balancing and data distribution (i.e.) the current request processing, whereas *IREA*_*Loc*_ represents the initial resource allocation for providing services to the users. The change detection for different wait times is presented in Fig 2.

The initial requests (i.e.) *rq*_*r*_ are classified as normal inputs for resource allocation. There are two outputs of the decision-making process (i.e.) whether there is a wait time or not. If there is a change in resource allocation time, then *WT* is computed. This computed *WT* is compared with *i* of the previous interval. Consequently if (*rq*_*p*_)_*i*_ is extracted (i.e.) (*rq*_*r*_−*rq*_*p*_)∀*i* ≠ 0 then paused requests are identified. Therefore the Δ*c* is observed for *FZ*(Δ) across various requests in the TSDF process (Refer to Fig 2). Thus, the temporary resource allocation *ϑ* is expressed as

ϑ=ΔC−WT (5)

By using Eq (5), the temporary resource allocation for the initial request and resource allocation for paused requests is computed for wait time. The TSDF employs a better method to find the optimal factor for load balancing and data distribution for preventing service delay and failure while joint resource allocation and distribution. Besides, the proposed framework defines the initial wait time *WT* minimizing condition and the final condition of the algorithm is to maximize low/high delay exhibiting distributions during continuous resource allocation. The wait time reduction *WT* is computed as

$$WT\leftarrow {rq}_{T}\times {rs}_{dT}$$ (6)

Such that,

$$\begin{array}{c} {rq}_{T}=\frac{1}{\pi }\sum_{-WT}^{i}\frac{D[{rq}_{p_{i}},\rho {\left({rq}_{p}\right)}_{i}]}{\Delta C}\\ and\\ {rs}_{dT}=\frac{1}{\pi }\sum_{-WT}^{i}\frac{I{REA}_{Loc}}{{rq}_{p_{i}}} \end{array}\}$$ (7)

**Fig 3:**
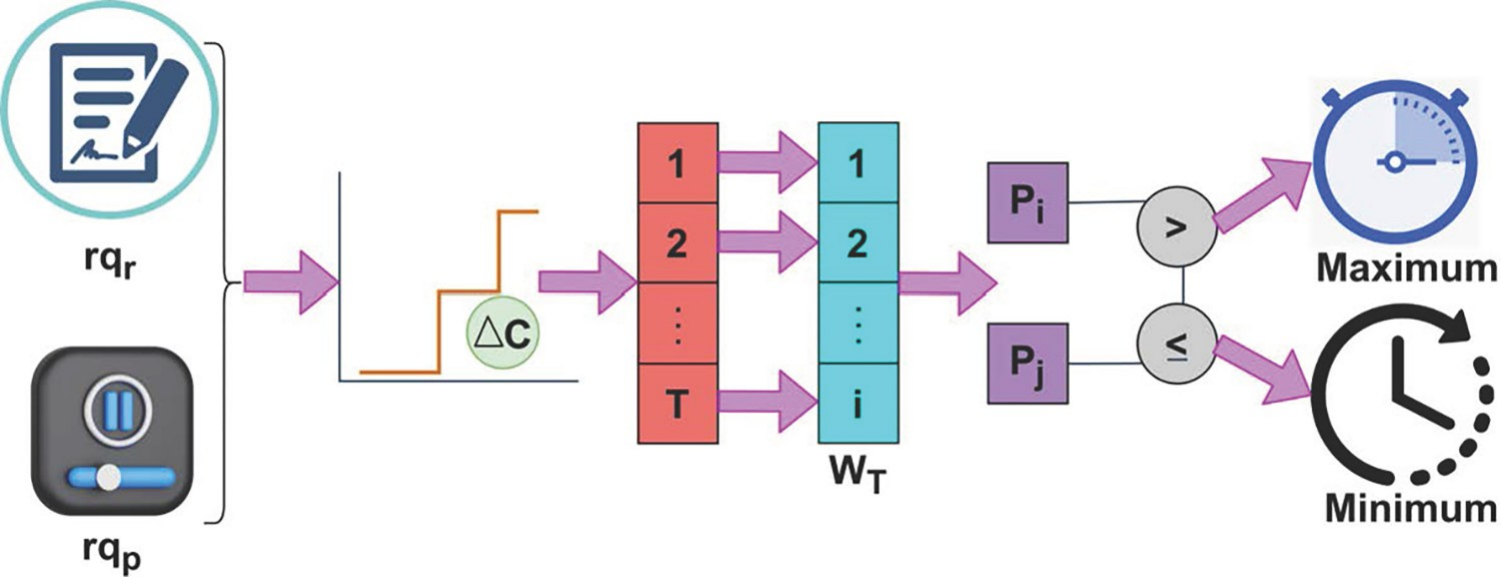
Minimum and maximum wait time request differentiation.

From the above Eq (6) and (7), the minimization of the wait time is described as a product of *rq*_*T*_ request processing time and *rs*_*dT*_ response delay time. Here, the temporary resource allocation for such requests is performed at the end of fuzzification outputs in maximum wait time. The process of fuzzification is explained below section. The fuzzy process for the minimum and maximum wait time differentiation process is illustrated in Fig 3.

The classified requests (i.e.) *rq*_*r*_ and *rq*_*p*_ are induced with Δ*c* through *FZ*(Δ) provided *D*(*rq*_*pi*_, *ρ*(*rq*_*p*_)_*i*_) generates *W*_*T*_. If the *w*_*T*_ is observed for distinguishable Δ*C* then resource allocation is concurrent. For concurrent resource allocation the *IREA*_*Loc*_ is performed through *FZ*(Δ). Based on the available *ρ*(*rq*_*p*_)_*i*_, the concurrent allocations reduce the *W*_*T*_ across *rq*_*r*_. Therefore the conditions *P*_*i*_ and *p*_*j*_ are validated if *p*_*i*_>*p*_*j*_ or *p*_*i*_≤*p*_*j*_ for which maximum/ minimum is classified accordingly (Refer to Fig 3).

### 3.2 Sequential resource allocation and distribution

After completing the paused request processing, the TSDF uses fuzzy logic for joint resource allocation and scheduling. The fuzzification is processed by combining the high and low delay exhibiting distributions for performing joint resource allocation of services to reduce the response delay. The fuzzification process is not effective for joint allocation in cloud computing. To solve this issue, the proposed TSDF utilizes fuzzy logic for providing space to minimize wait time occurred resource allocation, and distribution for appropriate load balancing. The FL is an optimization model based on identifying and segregating regular and paused requests for allocating space. Contrarily, with the existing cloud computing methods, the TSDF-FL combines all requests and performs the processes intending to improve the accuracy of load balancing and data distribution.

In the TSDF-FL model, the resources in the cloud environment are utilized for load scheduling and data distribution where the fuzzifications between regular and paused requests with minimum wait time. The fuzzy logic considers the following constraints to attain maximum high and low delay exhibiting distributions combined for joint resource allocation and distribution.

- The fuzzy logic first considered optimality determining features in the first level (i.e.) therefore, in the second level resource scheduling occurs.
- Resource allocation is relative to cumulative response delay. Therefore, the scheduling is performed with time-based for which the response delay is the optimal factor.
- The objective function is minimum time-varying requests are observed and fuzzified in the second level for further resource allocations.

In the fuzzy logic concept, the optimality determines features and time-based scheduling based on the objective function for joint allocation. To identify the time-varying requests from the second level for further allocation follow the distribution completed intervals for providing space for performing services. The similarity analysis is performed as the objective function in this proposed model. This similarity verification helps to discover the minimum wait time for resource allocation and distribution for sequential load balancing in the cloud environment. The sequence of resource allocation is to identify and classify the regular and paused requests for minimizing wait time and response delay. Fuzzy logic initializes the first user in the cloud environment with the help of providing services using TSDF. Afterward, fuzzification processes the scheduling and joint allocation with the help of objective function is the optimal solution. Fuzzy logic used similarity analysis of an objective function to accurately perform joint allocation and distribution. Next, fuzzy performs the resource scheduling according to the wait time and response delay.

Finally, fuzzy logic combines the high and low delay exhibiting distributions without impacting the scheduling for joint resource allocation. Thus, fuzzy logic forms observed time-varying requests for the identification of paused requests with higher data distribution and minimum wait time. As a result, the fuzzy logic enhances the load balancing rate and reduces the wait time for resource allocation and data distribution as compared to the existing methods. The fuzzification process first initializes the users in the cloud environment by distributing all optimality-determining features in the sample space *SaS* randomly for further resource allocation. The process of fuzzification is based on the varying load balancing and data distribution of services in cloud computing. The probability of scheduling occurrence *ρ*(*ϵ*) of determining features *μ*_*f*_ from the resource provider (*Rsc*_*prov*_)_*i*_ is validated with the use of an objective function *OB*_*Func*_ is expressed as

$$\rho (\epsilon ).\mu_{f}={OB}_{Func}+{\left({Rsc}_{prov}\right)}_{i}$$ (8)

**Fig 4:**
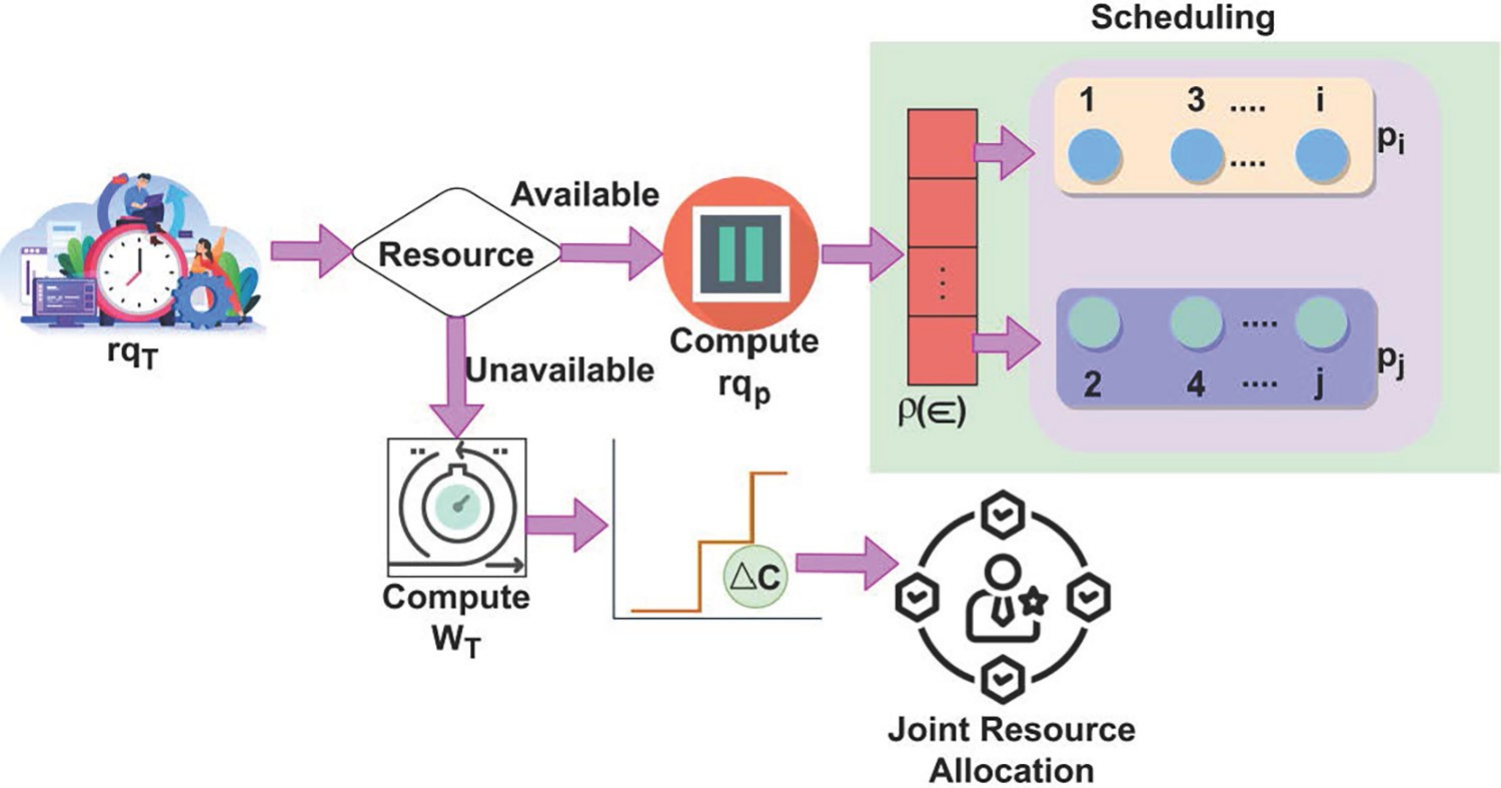
Scheduling for *rq*_*T*_.

Eq (8) follows the objective function where the probability of resource scheduling occurrence due to the maximum wait time is identified for reducing service delay and failures. The fuzzy logic considers an objective function to observe time-varying requests for joint resource allocation to improve its distribution for sequence processing of user requests to provide services. The scheduling process for *min* wait time for *rq*_*T*_ is illustrated in Fig 4.

The scheduling process for *rq*_*T*_ is performed in two distinct manners; *p*_*i*_ and *p*_*j*_ and Δ*C* based. In the first process *rq*_*p*_ is identified for allocation across *i* and *j*∀*ρ*(∈). This is required for suppressing maximum wait time such that the response (available) is completely used. In the unavailable case, the Δ*C* as in Fig 3 is performed for joint resource allocation. This allocation process is abrupt for preventing new *rq*_*p*_ failing *p*_*i*_≤*p*_*j*_ condition (Fig 4). The fuzzy logic utilizes similar verification to compute the same features in any requests, thereby finding space for resource allocation and data distribution. From that, the allocations used to determine the similarity between the features (*μ*_*f*_)_*i*_ and (*μ*_*f*_)_*j*_ are computed as

$$\omega_{same}\left({\left(\mu_{f}\right)}_{i},{\left(\mu_{f}\right)}_{j}\right)=\frac{{\left(\mu_{f}\right)}_{i}+{\left(\mu_{f}\right)}_{j}}{{\left\|{\left(\mu_{f}\right)}_{i}\right\|}^{2}+{\left\|{\left(\mu_{f}\right)}_{j}\right\|}^{2}-{\left(\mu_{f}\right)}_{i}*{\left(\mu_{f}\right)}_{j}}$$ (9)

Eq (9), the variable *ω*_*same*_ ((*μ*_*f*_)_*i*_, (*μ*_*f*_)_*j*_) refers to the similarity analysis between two features from which the scheduling occurrence is identified with less wait time. Thus, the maximum high and low delay exhibiting distributions based on features *ρ*(*μ*_*f*_)_*i*_ is determined as

$$\rho {\left(\mu_{f}\right)}_{i}=\omega_{same}\left({\left(\mu_{f}\right)}_{i},{\left(\mu_{f}\right)}_{j}\right)$$ (10)

From Eq (10), the joint resource allocation *JR*_*alloc*_ is performed based on scheduling occurrence ∁_*OC*_ between the determining features. Optimality in load scheduling and data distribution is an optimal factor with occurring resource scheduling ∁_*OC*_ is represented as

$$\rho ({∁}_{OC})=\alpha^{SaS}{rs}_{d}$$ (11)

Eq (11) computes the actual resource scheduling and allocation for wait time where the fuzzification between regular and paused requests is analyzed which reduces prolonged time. Then, the distribution completed intervals *δ*_*int*_ for such allocations is computed as

$$\delta_{int}=\delta_{{int}_{i}}exp(-\alpha^{SaS}{rs}_{d})$$ (12)

The above Eq (12) follows the distribution completed intervals between normal requests and paused requests for further resource allocations. Exponential distributions are frequently employed in queueing theory and performance modeling of systems with random arrivals, such as requests that arrive to a cloud scheduler. Here, constant cloud computing for joint resource allocation is performed with minimum wait time. The fuzzy logic utilizes the computation to allocate space for data distribution and load balancing is expressed as

$$\epsilon_{{\left(\mu_{f}\right)}_{i},{\left(\mu_{f}\right)}_{j}}=\frac{\sqrt{{\left\|{\left(\mu_{f}\right)}_{i}\right\|}^{2}+{\left\|{\left(\mu_{f}\right)}_{j}\right\|}^{2}-WT*{rs}_{d}}}{{rq}_{r_{i}}+\rho {\left({rq}_{p}\right)}_{i}}$$ (13)

Such that,

$${Max}_{WT}\forall \;rq\in T=\left\{(1-{\left\|{\left(\mu_{f}\right)}_{i}\right\|}^{2}+{\left\|{\left(\mu_{f}\right)}_{j}\right\|}^{2})\frac{{Max}_{{rq}_{dT}}}{{Min}_{{rq}_{dT}}}.-\alpha^{SaS}{rs}_{d}-({rq}_{r_{i}}+\rho {\left({rq}_{p}\right)}_{i})\right\},i\in rq$$ (14)

**Fig 5:**
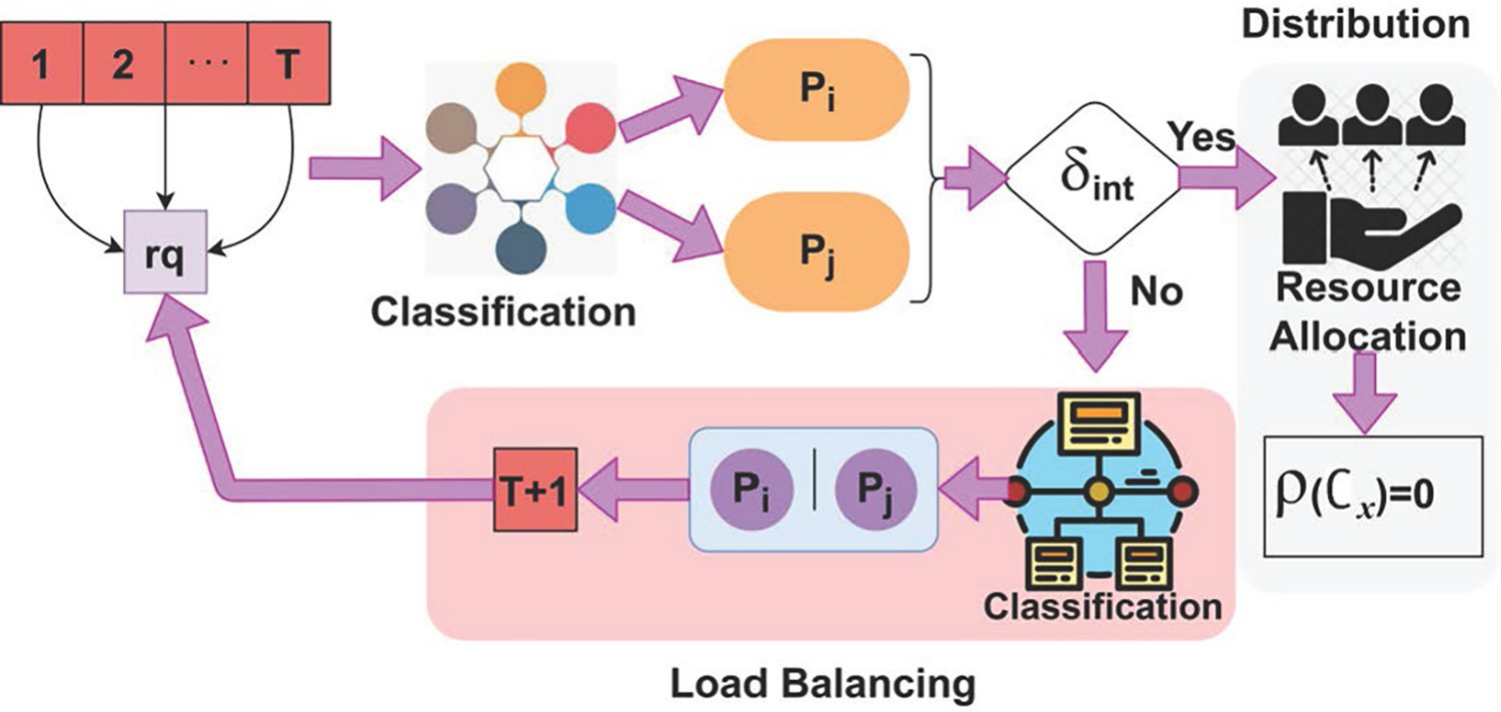
Load balancing for *rq*_*T*_.

The above Eq (13) and (14) computes the optimal load balancing rate with less response delay in the cloud environment. The mapping of load balancing and data distribution across different resource providers prevents prolonged time for the distribution completed interval *δ*_*int*_. The load balancing for *rq*_*T*_ is illustrated in Fig 5.

The load balancing is performed for (*T*+1) requiring *p*_*i*_ or *p*_*j*_ provided a classification from *δ*_*int*_ is performed. In the *p*_*i*_ performed. In the *p*_*i*_ or *p*_*j*_ estimation of the condition *ρ*(∁_*OC*_) = 0 is verified for completing the distribution process. On the contrary case if the resource allocations are not optimal then either *p*_*i*_ or *p*_*j*_ ∀*rq*_*T*_ (with *rq*_*p*_) is identified for (*T*+1)^*th*^ reallocation. Therefore the load is balanced in the distinguishable intervals (Fig 5). The resource allocation of the paused requests from the cloud environment is processed in two levels for joint resource allocation. For resource scheduling and joint resource allocation, the min/max wait time provides load balancing and resource allocation. This sequential process improves its data distribution and reduces the wait time and delay.

### 3.3 Self-analysis

**Fig 6:**
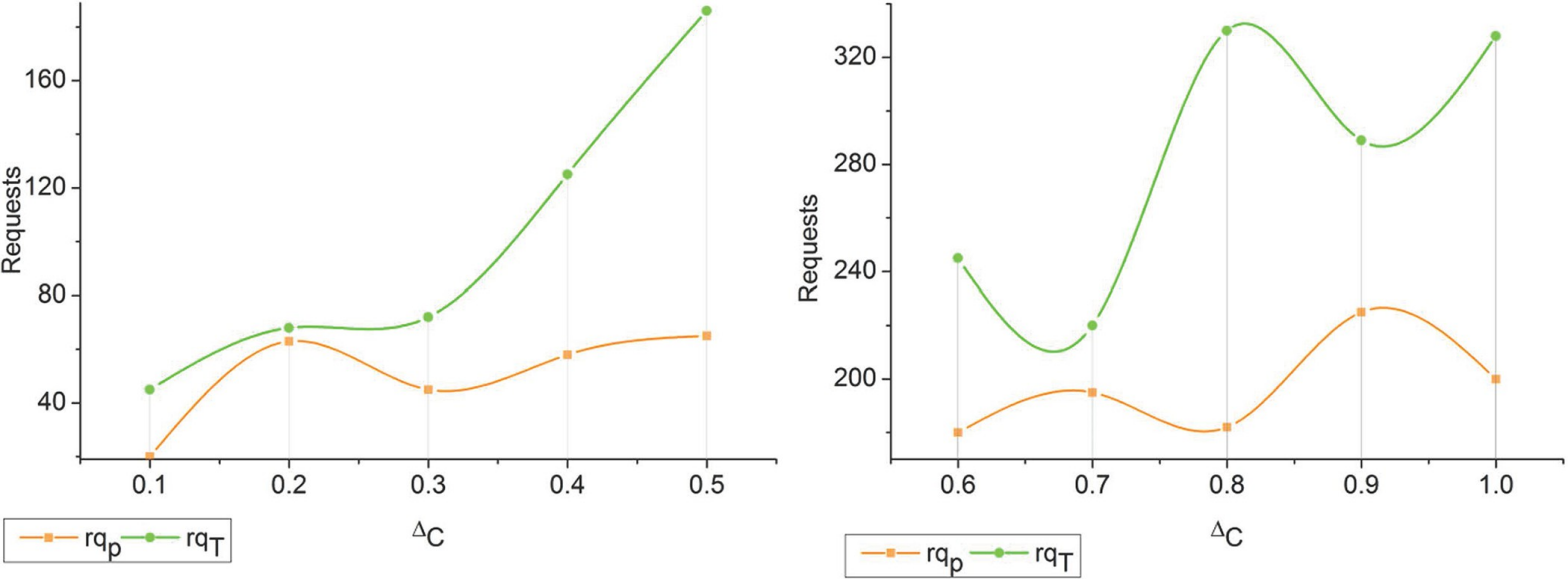
*rq*_*T*_ and *rq*_*p*_ analysis.

In Fig 6 the *rq*_*p*_ form the *rq*_*T*_ for Δ*C* is analyzed. This analysis is performed to verify if the allocation is optimal regardless of the service or resource providers.

**Fig 7:**
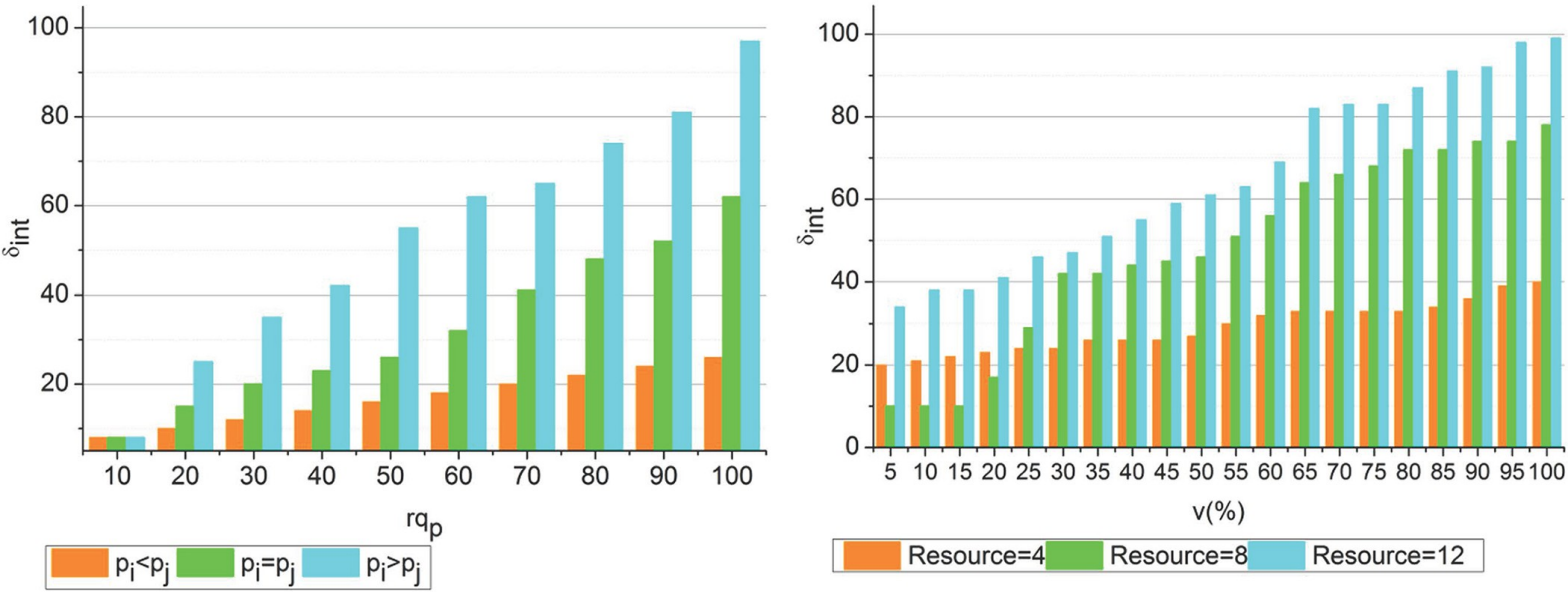
*δ*_*int*_ analysis.

Based on the fuzzy process the request classifications are performed. The conditional analysis for *p*_*i*_ and *p*_*j*_ are validated using data distribution and load balancing. The joint resource allocation is performed until *ρ*(∁_*x*_) is achieved to be less. Therefore the fuzzification is induced for *JR*_*alloc*_ such that *W*_*T*_ is confined. If the *W*_*T*_ is confined the consecutive allocations are optimal preventing further stagnancies. Thus the Δ*C* changes are reduced for the upcoming intervals. In this continuity the analysis for *δ*_*int*_ for the varying *rq*_*p*_ and *v* is presented in Fig 7.

The *rq*_*p*_ and *v* are inverse to each other; the *p*_*i*_ and *p*_*j*_ conditions influence the *δ*_*in*_ ∀*rq*_*p*_. The fuzzy process confines *p*_*i*_>*p*_*j*_ to *p*_*i*_ =*p*_*j*_ or *p*_*i*_<*p*_*j*_ using *FZ*(Δ)∀*ρ*(∈). This case is optimized using *ρ*(*μ*_*f*_)_*i*_ and *ρ*(∁_*x*_) = 0 achieving conditions. Therefore the completed intervals are high for the fuzzy optimized case, contrarily, if *v* increases for the number of resources then *ε*(*μ*_*f*_)_*i*_ is high and hence the balance increases. Therefore *FZ*(Δ) is increased in the alternating (*T*+1) achieving high *δ*_*int*_ (Fig 7).

## 4 Results and discussion

The results and discussion section presents a comparative analysis using the metrics data distribution, resource scheduling, resource allocation, waiting time, response delay, and load handling rate. The evaluation metrics used in the research study evaluates the set of performance measures designed to assess the effectiveness and efficiency of the proposed TSDF-FL model for cloud computing resource management. Each metric serves a specific purpose and provides insights into different aspects of the model performance. The resource providers (1 to 14) and the number of user/ application requests (20–320) are the variables used. The methods RACER [21], MaOEA-AES [31], and CSC-ACO [19] are used alongside the proposed method for verifying the proposed method’s consistency.

The user defined parameters are variables set by the cloud users that includes wait time, load handling rate, time-based computation defined along with the request for efficient resource utilization. The parameters are adopted for preventing service delay and failure rate in a cloud environment. The initial user request processing is selected with the FL observation of preventing maximum wait time to compute the output based on resource allocation and load distribution with less time consumption. The feature parameter lies in the temporary resource allocation and later these user defined features are analyzed using FL for allocating space service. Also based on the request types from the users like normal or paused ensures the load balancing and data distribution is chosen. The user defined features related to requests are optimized with the similarity analysis of features and wait time is reduced and high data distribution is obtained by these users.

### 4.1 Data distribution

Data distribution metric evaluates how well the model distributes data and workloads among different resource providers. In order to make certain that resources are used effectively and that no one provider is overburdened while others are underutilized, data distribution is essential in cloud computing. A reliable method for distributing data aids in balancing the load and guards against resource constraints.

**Fig 8:**
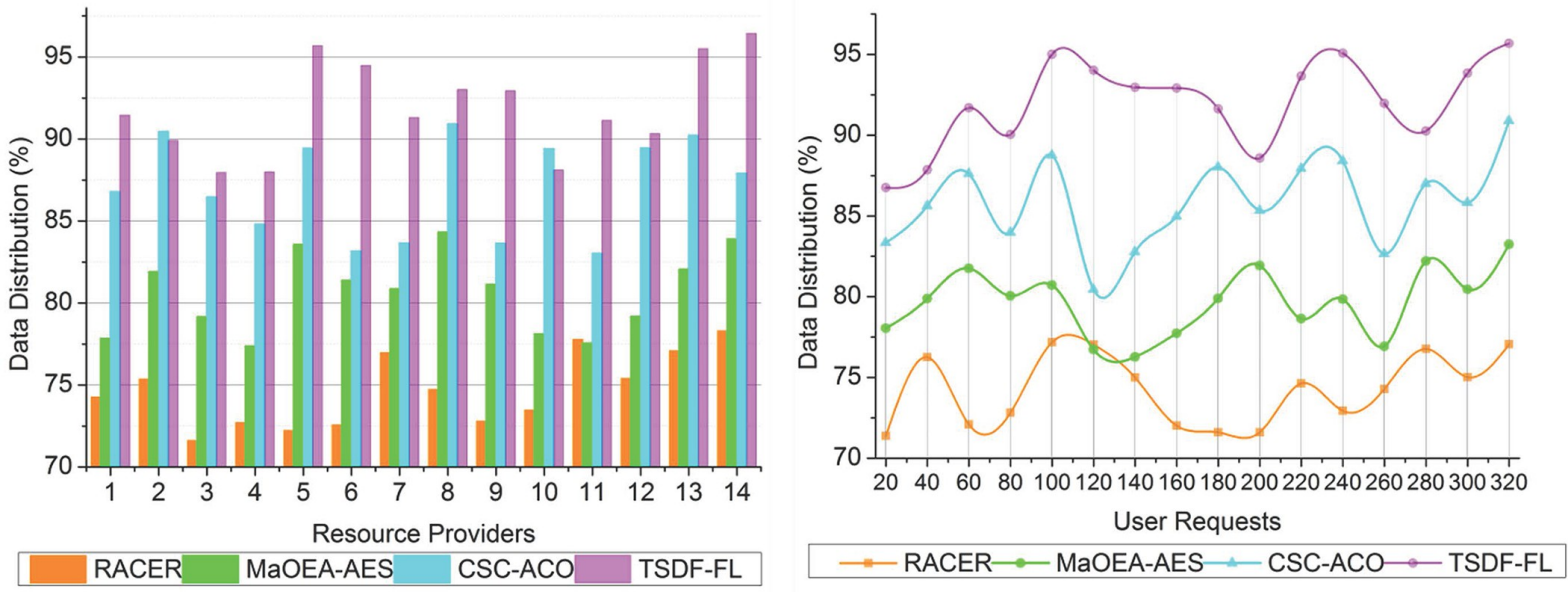
Data distribution.

In Fig 8, the optimality in load balancing and data distribution is paused through TSDF using FL for reducing the maximum wait time in cloud computing to improve the data distribution and resource allocation. The variations in data distribution are most likely a result of fluctuating resource availability and incoming user requests. To optimize data dissemination, the TSDF-FL model applies temporary resource allocation and fuzzy logic. Variations between request types and the availability of resources over time may be to blame for fluctuations. The two-level scheduling and distribution do not provide continuous services for a prolonged time. Using different technologies and resource scheduling from the paused request is analyzed for improving load handling rate in distribution completed intervals. The fuzzy logic determines features for identifying and segregating normal requests and paused requests using the condition $FZ\sum_{WT}{rq}_{p_{i}}ln\;\rho {\left({rq}_{p}\right)}_{i}$. The initial user request processing is to enhance the resource allocation and distribution for computing initial output wherein less time utilization due to maximum wait time is observed. These aforementioned issues are addressed using TSDF-FL and joint resource allocation is performed for improving data distribution based on the load scheduling across different resource providers, preventing response delay. Therefore, the similarity analysis of features is to reduce wait time and high data distribution is achieved by the following users.

### 4.2 Resource scheduling

The Resource Scheduling measure expresses how many requests from users or applications are allocated for every resource provider. The effectiveness of resource scheduling governs how resources are distributed among jobs. An increase in requests per provider may signify better resource management and less idle capacity.

**Fig 9:**
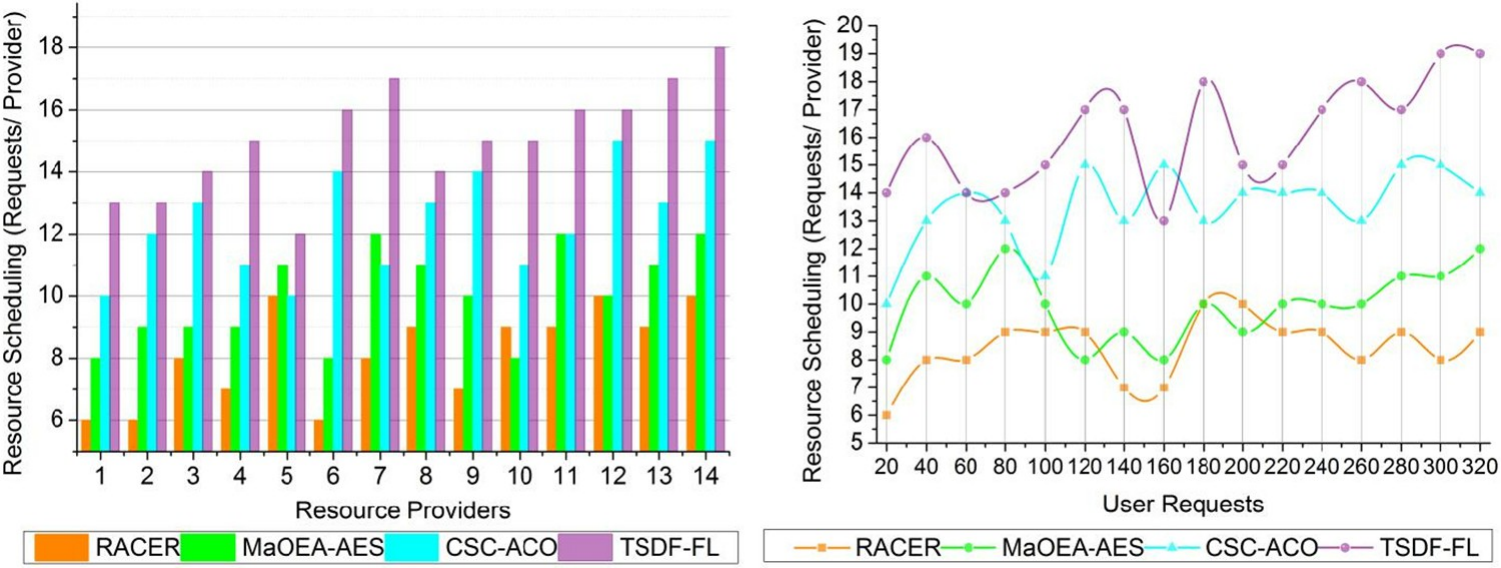
Resource scheduling.

The two-level scheduling and distribution are processed for the users based on normal requests and paused requests ensuring load balancing and data distribution for joint resource allocation, the maximum wait time due to high/low delay exhibiting distributions. The objective function of the TSDF-FL model is performed with unattended requests for the sequence based on different resource providers in a cloud environment. The probability of scheduling occurrence *ρ*(*ϵ*) with determining features *μ*_*f*_ is processed for providing services to the resource provider as represented in Fig 9. The variations of values in the above-mentioned graph brought on by the fluctuating nature of user demands, the volume of demands, and the resource providers’ capability. A combination of request type and their availability of resources, which can change over time, TSDF-FL seeks to allocate resources in an efficient manner. In this proposed model satisfies high resource scheduling and data distribution using fuzzy logic for which the cumulative response delay is identified and reduced with the TSDF-FL model. In this fuzzy logic improving the load handling rate and reducing wait time for processing joint resource allocation until processing all requests in a cloud environment at different time intervals, prevents failures and service delays. Therefore, the load scheduling and data distribution are processed through fuzzy logic maximizing joint resource allocation and resulting in minimum wait time achieving high resource scheduling with TSDF.

### 4.3 Resource allocation

Resource Allocation statistic evaluates how efficiently resources are distributed in order to satisfy user or application requirements. For activities to be performed on time and for resources to not be wasted, effective resource allocation is essential. Increased resource allocation values denote increased effectiveness.

**Fig 10:**
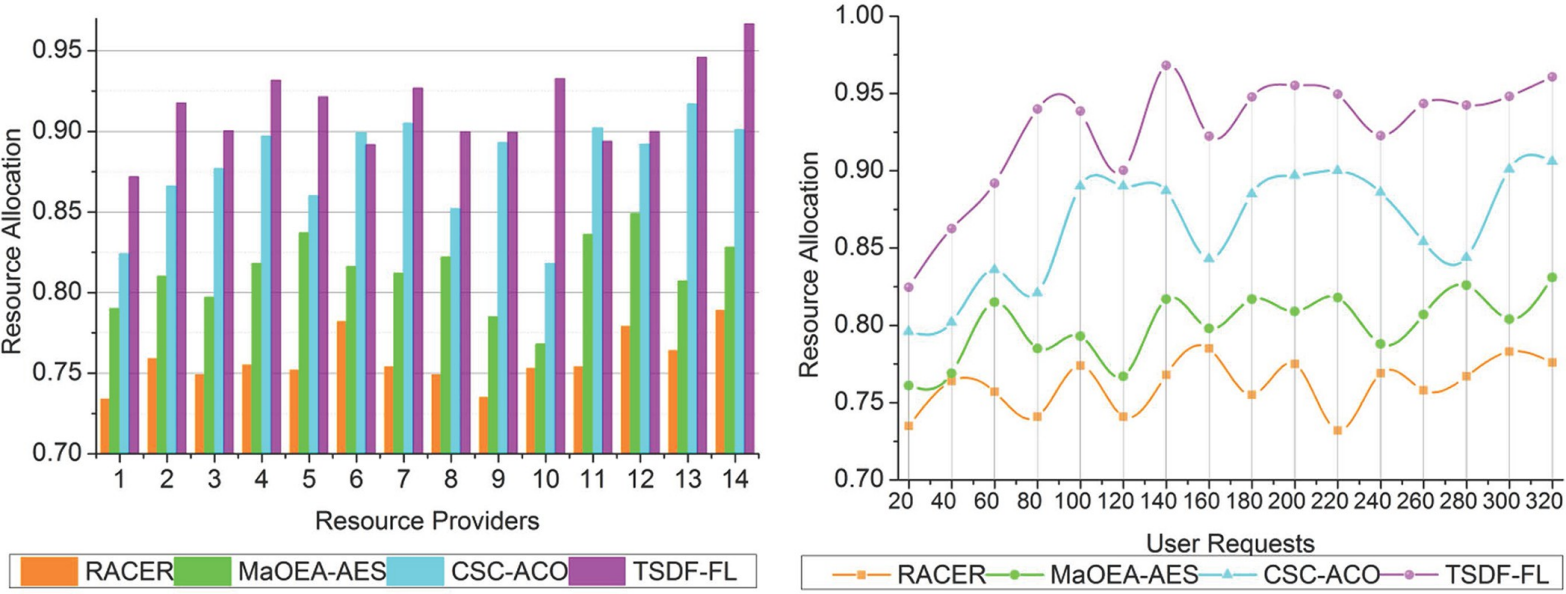
Resource allocation.

This proposed model achieves high resource allocation and load handling rate depending on the fuzzification process and similarity feature analysis for identifying and classifying normal requests and paused requests for proving accurate services (Refer to Fig 10). The varying requests and their unique resource needs may be the cause of the changes in resource allocation.

Resource allocation is optimized by TSDF-FL using FL and feature analysis, but this can change depending on the particular requests being handled. The sequential resource allocation and scheduling are performed using TSDF-FL model and the current request processing compares with an existing method for minimizing wait time. The fuzzy logic first initializes the user’s request by distributing the features for temporary resource allocation for such requests; this process prevents maximum wait time. Sequential resource allocation is performed based on different fuzzification processes for load balancing and data distribution for identifying the response delay. The fuzzification process is performed with varying load balancing and data distribution for a prolonged time between regular and paused requests at different time intervals. Therefore, the optimal factor is achieved in this model based on observing maximum wait time and the addressed issues are solved using fuzzy logic therefore, the resource allocation and scheduling are high and the load balancing rate also increases.

### 4.4 Waiting time

The amount of time an application or user’s request must wait in the queue without receiving the resources it needs is measured as waiting time. For cloud services to be responsive and effective, waiting times must be minimized. Faster resource access and higher user satisfaction are both made possible by shorter wait times.

**Fig 11:**
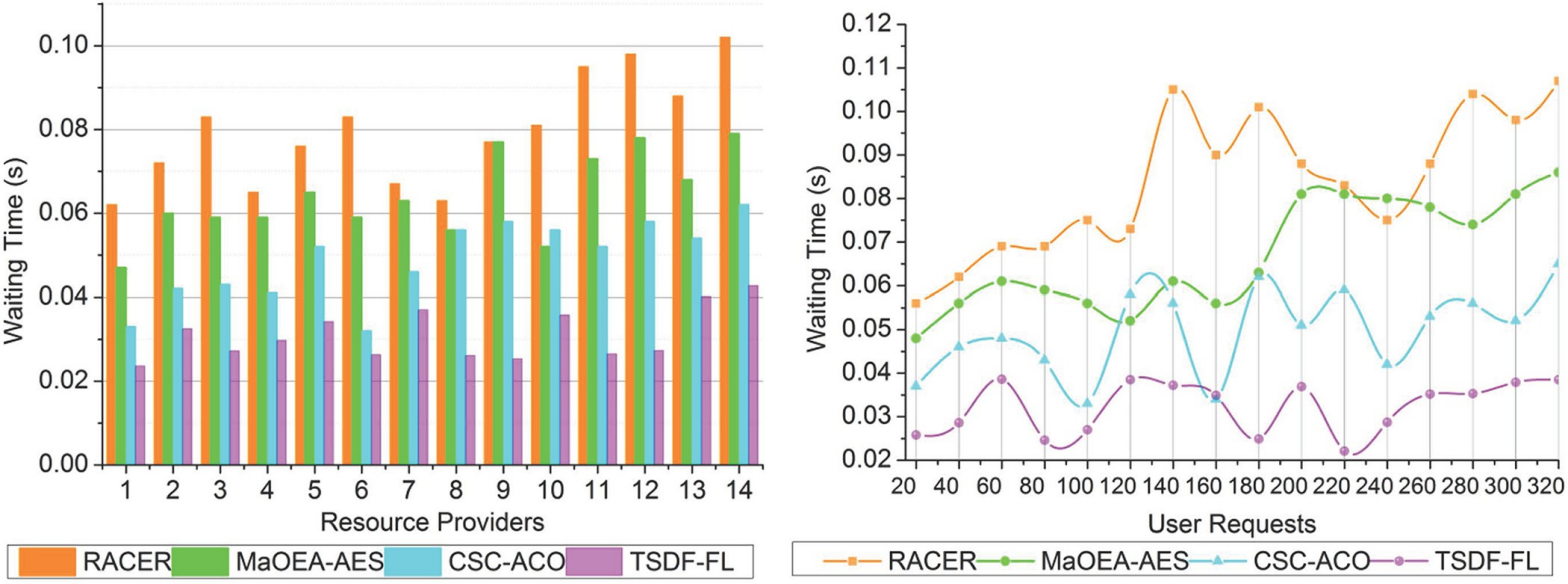
Waiting time.

The user request processing in the cloud environment improves the performance of data distribution and load balancing based on user requests and service response from the end-users and its wait time for processing at different time intervals is depicted in Fig 11. Fluctuations like an increase or decrease in waiting times may happen as a result of variations in the volume of requests that arrive and the cloud system’s processing power. Although TSDF-FL optimizes the distribution of resources and load balancing, there may still be fluctuations in waiting times. In this resource allocation in the cloud environment is difficult, the service delay and failure is occurred in that platform such that the maximum wait time is satisfied by the users for scheduling. After completing the distribution, the determining features are administered using fuzzy logic for allocating space and providing services. The response delay is identified at the time of paused request processing in the cloud environment for load balancing and data distribution. In this proposed model, the resource allocation relies on connected users in cloud computing for ensuring wait time and space complexity for distribution is analyzed for improved optimality of unattended requests processing. The wait time minimization is performed to satisfy a high load handling rate and prevents service delay. Hence, the waiting time is less compared to the other factors in this proposed model.

### 4.5 Response delay

Response delay gauges how long it takes for a user or application request to get an answer or finish running. Real-time or minimal latency services must be offered with the least amount of response delay possible. Greater responsiveness and efficiency in resource distribution are indicated by lower response latency values.

**Fig 12:**
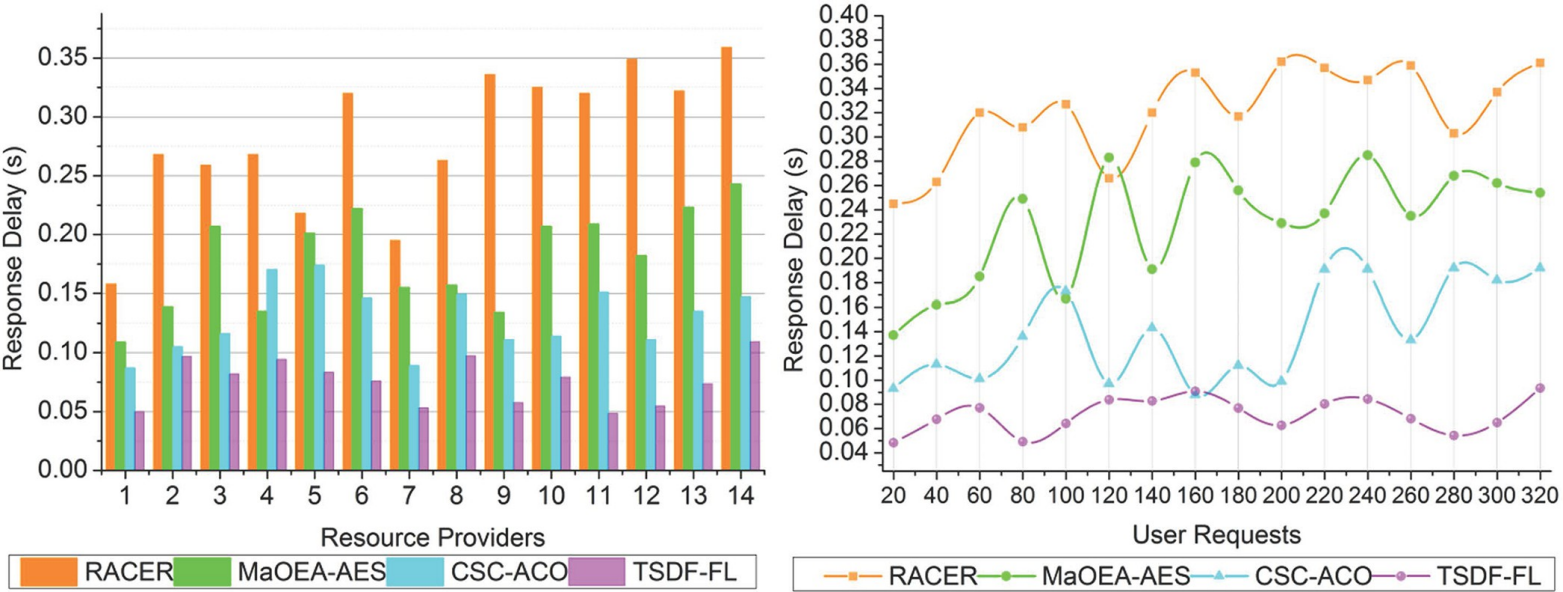
Response delay.

The service failures/delay identification in this cloud environment with fuzzy logic concepts is to improve the joint resource allocation for a huge number of requests and classify the user requests based on regular and paused using the TSDF-FL model for reducing response delay. The user request is processed in the cloud environment, and the incremental service failures and response delay is identified across different resource provider as represented in Fig 12. Response times can vary depending on the state of the network, the difficulty of the request, and the amount of data load on the cloud service. Although TSDF-FL uses fuzzy logic to reduce response delays, they can change based on the nature of the requests and the state of the system. In this proposed model, the response delay at the time of resources allocation and scheduling for the connected users in a cloud platform is analyzed for load balancing. From the condition *rq*_*WT*_>*rq*_*dT*_ is computed for identifying service connections and resource management that augments joint resource allocation. The continuous unattended request processing using fuzzy logic, and the wait time for resource allocation and scheduling in the cloud environment are computed for achieving optimal factors. Based on the first-level and second-level output, the time-varying requests are fuzzified for further processing using Eqs (6), (7), (8), (9), and (10) computations. From this request classification in cloud computing, the response delay is less compared to the other factors.

### 4.6 Load handling rate

The effectiveness with which resource providers manage peak demands is measured by this indicator. Provider in cloud computing must manage varying workloads. An increased load handling rate shows that service providers can effectively handle periods of high demand, assuring system stability and dependability.

**Fig 13:**
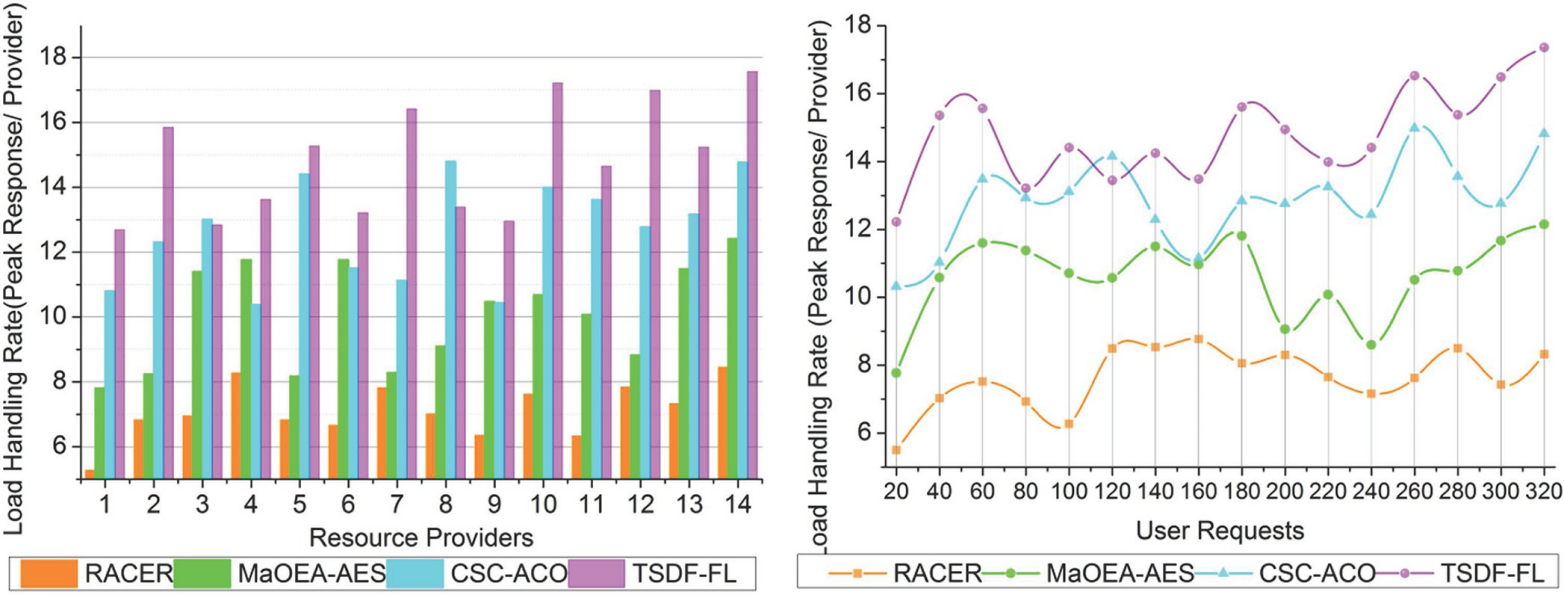
Load handling rate.

This TSDF-FL model achieves a high load handling rate across different resource providers in distribution completed intervals to maximize the optimal resource allocation and scheduling for reducing the wait time and response delay (Refer to Fig 13). The quantity and caliber of incoming requests can fluctuate, which can cause variations in load handling rate. In order to improve load handling, TSDF-FL optimizes resource allocation and distribution. However, it reacts dynamically to changing needs. The service failures and high/low delay exhibiting distributions is mitigated through fuzzy logic for joint resource allocation for reducing the maximum wait time due to time-varying requests processing. The user requests services provided using fuzzy logic and processing of accumulated data through the TSDF model. The observed user requests are processed for performing appropriate load balancing and data distribution with the time-based computation for addressing the service failures and delays to improve the load handling rate. The observed user requests are classified and analyzed for reducing waiting time and response delay. Similarly, the optimality-determining features are compared with existing features for improving the data distribution. Therefore, the optimality in load balancing and data distribution from the paused requests depends on the fuzzification process for further resource allocation and distribution. Hence, the high load handling rate is achieved by the fuzzy logic. Tables 1 and 2 present the comparative analysis results for the varying resource providers and user requests.

**Table 1:** Comparative analysis results (resource providers).

Metrics	RACER	MaOEA-AES	CSC-ACO	TSDF-FL
Data Distribution (%)	78.3	83.9	87.91	96.436
Resource Scheduling (Requests/ Provider)	10	12	15	18
Resource Allocation	0.789	0.828	0.901	0.9666
Waiting Time (s)	0.102	0.079	0.062	0.0427
Response Delay (s)	0.359	0.243	0.147	0.1093
Load Handling Rate (Peak Response/ Provider)	8.45	12.42	14.78	17.568

#### 4.6.1 Findings

The proposed model improves data distribution, resource scheduling, allocation, and load handling rate by 13.07%, 10.48%, 12.73%, and 11.4% respectively. The waiting time and response delay are reduced by 7.8% and 9.36% respectively.

The evaluation in Table 1 is based on the actions and output of the cloud resource service providers. This table’s rows each reflect a distinct framework, while its columns each represent a particular performance statistic. The table shows the way each framework performs at various resource provider tiers. There could be one to fourteen resource providers. The efficacy of the framework in relation to the traits and capacities of cloud resource providers is principally assessed in Table 1. It evaluates how efficiently different frameworks deploy resources to fulfil incoming requests. The evaluation in Table 2 is based on requests submitted to the cloud infrastructure by users or applications. Each column refers to a certain performance indicator, and each row defines a different framework. The table displays each framework’s performance under various user request levels. 20 to 320 different user requests are possible.

**Table 2:** Comparative analysis results (user requests).

Metrics	RACER	MaOEA-AES	CSC-ACO	TSDF-FL
Data Distribution (%)	77.06	83.24	90.89	95.688
Resource Scheduling (Requests/ Provider)	9	12	14	19
Resource Allocation	0.776	0.831	0.906	0.9607
Waiting Time (s)	0.107	0.086	0.065	0.0385
Response Delay (s)	0.361	0.254	0.192	0.0934
Load Handling Rate (Peak Response/ Provider)	8.32	12.15	14.82	17.358

#### 4.6.2 Findings

The proposed model improves data distribution, resource scheduling, allocation, and load handling rate by 11.96%, 12.87%, 12.3%, and 11.19% respectively. The waiting time and response delay are reduced by 9.21% and 10.88% respectively. Table 3 describes the list of abbreviations used in this research.

**Table 3:** List of abbreviations.

Abbreviation	Definition
Two-Level Scheduling and Distribution Framework	TSDF
Fuzzy Logic	FL
Fuzzy-Optimized Data Management	FDM
Clustering-Based Multiple Objective Dynamic Load Balancing Technique	CMODLB
Evolutionary Computation	EC
Quality of Service	QoS
Ant Colony Optimization	ACO
Cloud Service Composition	CSO
Resource Allocation Control Engine with Reinforcement Learning	RACER
Particle Swarm Optimization	PSO
Interval Type-2 Fuzzy C-Means	IT2FCM
Adaptive Iterated Local Search	AILS
Dynamic Inertia Particle Swarm Optimization	DI-PSO
Multi-Objective Scheduling Algorithm using Fuzzy Resource Utilization	FR-MOS
Software-Defined Cloud Manufacturing	SDCM
Software-Defined Network	SDN
Many-Objective Evolutionary Algorithm using Adaptive Environment Selection	MaOEA-AES
Internet of Things	IoT
Analysis Of Variance	ANOVA

### 4.7 Analysis of Variance (ANOVA) analysis

The FL and two-stage distribution framework of the TSDF-FL algorithm are used to optimize data distribution. The considerable difference in measurements for data distribution implies that it succeeds in achieving its objectives of enhancing data dissemination, avoiding resource bottlenecks, and guaranteeing effective resource utilization. The analysis of a sizable performance difference between the approaches, including the TSDF-FL algorithm, in the ANOVA assessment for Data Distribution metric. The p-value shows that at least one method’s data distribution performance differs noticeably from that of the others in terms of user request. Based on the results of the ANOVA analysis, it is suggested that a cloud service provider employ the TSDF-FL approach to resource management in order to increase performance and resource utilization and, ultimately, deliver better services to its users. The statistical software libraries with SciPy in a Python are used as a tool for calculating data distribution metric.

By taking the hypothetical example the Anova measure yields the significant result with a very low p-value (0.0002) that is much less than the typical significance level of 0.05. Then the rejection of null-hypothesis (H0) is concluded with the significant difference in data distribution among the comparison methods. The analyzed p-value is <0.05 with the F-statistic measures as 13.21 with the sum of squares and the degree of freedom values as 39 for each user request group. For the number of users in the cloud, variation in performance of data distribution metric between different methods is analyzed. Within groups of TSDF-FL approach analyzes the performance of different users varies when there are more of fewer users in the cloud environment.

The TSDF framework seeks to offer a comprehensive method for efficiently distributing workloads and data across diverse cloud resources. By utilizing a two-level hierarchical architecture and harnessing fuzzy logic, this approach has the potential to be implemented in various cloud computing settings beyond the specific configuration examined in this work. The dynamic scheduling and continuous load balancing provided by the TSDF can optimize resource utilization and meet quality of service requirements in public cloud environments with different tenants and fluctuating workloads. The capability to manage diverse resources makes it applicable to hybrid clouds that integrate both private and public infrastructure. The TSDF’s data localization and load distribution elements can reduce data transfer overheads and computational bottlenecks in performance-sensitive applications such as big data analytics, streaming processing, or computational clusters. The proposed methodology is also applicable to upcoming fog-cloud systems. The hierarchical design aligns well with the scattered edge nodes and centralized cloud of the fog, facilitating strategic allocation of workloads based on considerations such as latency, bandwidth, and processing requirements. However, the extent to which the findings may be applied to varied workload characteristics, scales, and levels of heterogeneity depends on the strength and reliability of the fuzzy rulesets. Customizations may need to be made to the fuzzification modules to suit the particular cloud environment and application scenarios. under general, the TSDF demonstrates potential as a flexible scheduling solution for effective resource management under different cloud paradigms and user requirements.

The TSDF’s primary objective is to enhance load balancing and data distribution in diverse situations. However, it may encounter obstacles in effectively predicting and adjusting to extremely dynamic and bursty workload patterns. Under conditions of excessive load fluctuation, the centralized scheduling system may become a single point of performance bottleneck. Accurately determining the specific resource needs of various types of workloads and their data interdependencies is a difficult task. In addition, the constant monitoring and rescheduling required in the second level for workloads that change over time may lead to higher latencies, particularly in fog environments with limited processing capacities at the edge nodes.

## 5 Conclusion

A Two-level Scheduling and Distribution Framework using Fuzzy Logic (TSDF-FL) model is designed for improving its distribution and reducing the wait time. The optimality-determining features are extracted from the different technologies and resource scheduling in the cloud platform with maximum wait time and contain several time-varying requests. Hence, wait time reduction is required for data distribution, and load balance scheduling is paused at the time of observing unattended requests for a prolonged time to identify service delays and failures. The TSDF is to perform joint resource allocation and scheduling for processing such requests. First, TSDF observed input data from the heterogeneous services, data, and users in the cloud environment. Then, TSDF processes the regular requests from that cloud environment and performs the load scheduling and data distribution (i.e., initial solution), and then allocates resources (i.e., for providing services) with the application using fuzzy logic. The limitation of the current study involves running complex algorithms like FL for resource allocation and scheduling may require significant computational resources. The important factor is to assess the resource requirements and potential bottlenecks that affects the dynamic workload capacity of the proposed TSDF. The proposed model improves data distribution, resource scheduling, allocation, and load handling rate by 13.07%, 10.48%, 12.73%, and 11.4% respectively. The waiting time and response delay are reduced by 7.8% and 9.36% respectively. In future, enhance the TSDF framework by incorporating completely decentralized scheduling methods that utilize multi-agent systems or distributed consensus algorithms. This will enhance scalability and fault-tolerance in large-scale cloud environments.

## Abbreviations

Symbol: Explanation
As *CE*^*u*^: Set of users
*rq_r_*: Regular request
*ρ*(*rq*_*p*_)_*i*_: Load balancing
Δ: Optimality
*FZ*: Constant fuzzification process
*WT*: Waiting time reduction
Δ*C*: Changes in features
$D\left[{rq}_{p_{i}},\rho {\left({rq}_{p}\right)}_{i}\right]$: Different requests
*IREA_Loc_*: Initial resource allocation for providing service to users
*ϑ*: Temporary resource allocation
*rs_dT_*: Response delay time
*rq_T_*: Request processing time
*OB_Func_*: Objective function
*ρ*(*ϵ*): Probability of scheduling occurrence
*μ_f_*: Determining features
*ω_same_*: Similarity between features
∁_*OC*_: Occurring resource scheduling
*δ_int_*: Completed interval
