# Expression of concern: Optimizing load scheduling and data distribution in heterogeneous cloud environments using fuzzy-logic based two-level framework

**DOI:** 10.1371/journal.pone.0345146

**Published:** 2026-03-18

**Authors:** The PLOS One Editors

The *PLOS One* Editors issue this Expression of Concern because this article [1] was identified as one of a series of submissions for which we have concerns about the peer review process. Readers are advised to interpret the article [1] with caution.

The Editors have no evidence of any author involvement in the peer review concerns.
